# An Erythrocyte Membrane-Associated Antigen, PvTRAg-26 of *Plasmodium vivax*: A Study of Its Antigenicity and Immunogenicity

**DOI:** 10.3389/fpubh.2020.00148

**Published:** 2020-04-28

**Authors:** Liping Fan, Jinxing Xia, Jilong Shen, Qiang Fang, Hui Xia, Meijuan Zheng, Jin-Hee Han, Eun-Taek Han, Bo Wang, Yuanhong Xu

**Affiliations:** ^1^Department of Clinical Laboratory, the First Affiliated Hospital of Anhui Medical University, Anhui, China; ^2^The Key Laboratories of Parasitology and Zoonoses Anhui and Department of Parasitology, Anhui Medical University, Anhui, China; ^3^Department of Microbiology and Parasitology, Bengbu Medical College, Anhui, China; ^4^Anhui Key Laboratory of Infection and Immunity, Bengbu Medical College, Bengbu, China; ^5^Department of Medical Environmental Biology and Tropical Medicine, School of Medicine, Kangwon National University, Chuncheon, South Korea

**Keywords:** malaria, *Plasmodium vivax*, tryptophan-rich antigens, immunogenicity, vaccine candidate

## Abstract

**Background:** *Plasmodium* tryptophan-rich (TR) proteins have been proposed as potential vaccine candidate antigens. Among them, *P. vivax* tryptophan-rich antigens (PvTR-Ags), which have positionally conserved tryptophan residues in a TR domain, are highly antigenic in humans. Several of these antigens, including PvTRAg-26, have exhibited erythrocyte-binding activities.

**Methods:** Subclasses of IgG antibodies against PvTRAg-26 were detected by enzyme-linked immunosorbent assay in 35 *P. vivax* infected patients and mice immunized with the recombinant antigen to characterize its antigenicity and immunogenicity. Moreover, the antigen-specific immune responses and Th1/Th2-type cytokine patterns of splenocytes from the immunized animals were determined *in vitro*. The subcellular localization of PvTRAg-26 in ring-stage parasites was also detected by indirect immunofluorescence assay.

**Results:** The IgG1 and IgG3 levels in *P. vivax*-infected patients were significantly higher than those in uninfected individuals. In the PvTRAg-26-immunized mice, elevated levels of antigen-specific IgG antibodies were observed, dominated by the IgG1 subclass, and Th1-type cytokines were remarkably increased compared with Th2-type cytokines. Additionally, the subcellular location of the PvTRAg-26 protein was closely associated with the caveola-vesicle complex on the infected-erythrocyte membrane in the early ring stage of *P. vivax*.

**Conclusions:** PvTRAg-26, a *P. vivax* TR antigen, with high antigenicity and immunogenicity, induces Th1-cytokine response and increases production of IgG1 antibodies. This immune profiling study provided a substantial evidence that PvTRAg-26 may be a potential candidate for *P. vivax* vaccine development.

## Introduction

*Plasmodium vivax* is the predominant malaria parasite epidemic in Asian and South American countries, which affects millions of people each year (1). In most cases, the parasite causes benign malaria. However, it may give rise to a severe, even fatal infection (2–4). It has been well established that malaria parasites have presented relative resistance to commonly used anti-malarial drugs. Thus the identification of novel anti-malarial drugs and the development of vaccines are urgently needed for effective control of the disease.

In a long time, development of *P. vivax* vaccines has been hindered by the absence of a continuous *in vitro* culture system and low-level parasitemia of patients (5). Therefore, the majority of *P. vivax* vaccine studies are focused on orthologous antigens of *P. falciparum*; circumsporozoite surface proteins (CSPs), thrombospondin-related adhesive protein (TRAP) of the pre-erythrocyte stage, apical membrane antigen-1 (AMA-1), Duffy-binding protein (DBP), rhoptry-associated proteins, merozoite surface proteins of the erythrocyte stage, and Pvs25 and Pvs28 from sexual stage of the parasite (6–10). Among them, only three antigens of CSPs, TRAP, and Pvs25 of *P. vivax* have been extensively investigated in clinical vaccine trials (11). However, the novel practical vaccine molecules of *P.vivax* remain undiscovered.

*Plasmodium* tryptophan-rich antigens (TR-Ags) have been proposed as a group of potential vaccine candidates. The TR-Ags were first identified in the murine malaria parasite of *P. yoelii*. Mice immunized with the recombinant TR-Ags produced highly protective immunity against *P. yoelii* infection (12, 13). Similarly, TR-Ags of *P. falciparum* could inhibit the invasion of erythrocytes by its merozoites (14). The genome of *P. vivax* encodes more TR-Ags than that of any other *Plasmodium* species. So far, fifteen TR-Ags have been found to be able to evoke significant cellular and humoral immune responses in *P. vivax-*exposed individuals (15). A recent study showed that TR-Ags could bind to normal human erythrocytes and the process could be inhibited by the sera of malaria patients (16). Our previous research also revealed that the conserved TR motifs exist in most PvTR-Ags which have high antigenicity in *P. vivax* infection, even in patients from low-endemic regions. We recently demonstrated that there are five proteins that are associated with the caveola-vesicle complex (CVC) structure, a unique structure of *P. vivax*-infected erythrocytes (17).

Among the five PvTR-Ags, PvTRAg-26 is an erythrocyte-binding protein (16). Although the antigenicity of PvTRAg-26 was partially tested in the previous study in *P. vivax* patients, the nature of the IgG subclass response to PvTRAg-26 in patients and the immunogenicity of PvTRAg-26 remain unclarified either *in vitro* cell experiments or *in vivo* animal experiments. Moreover, the membrane-associated subcellular localization needs to be investigated. In the present study, we tested the antigenicity and immunogenicity of PvTRAg-26 in the serum samples collected from symptomatic *P. vivax* patients as well as PvTRAg-26 immunized mice. Total IgG antibody and its subclasses were detected in the blood and the antigen-specific immune response and Th1/Th2-type cytokines of splenocytes were measured. Additionally, the subcellular localization of the PvTRAg-26 antigen on the membrane of *P. vivax-*infected erythrocytes was also performed.

## Materials and Methods

### Human Serum Samples

Serum samples of 35 malaria patients were collected in the hospitals of Bengbu and Hefei, Anhui province of China, all of them showing positive *P. vivax* parasite by microscopy. Simultaneously, fifteen serum samples of the individuals from malaria non-endemic areas were taken as control. The positive or negative sera were confirmed by both microscopy and nested PCR methods (18).

### Expression and Purification of Recombinant PvTRAg-26

Genomic DNAs were prepared from *P. vivax* isolates and used as templates for PCR amplifications. PvTRAg-26 coding genes were amplified with primers of PvTRAg-26-F (5′-CCTTCACTTATAGATAAGTACGATGCT-3′) and PvTRAg-26-R (5′-TTATATTTTTGAATTCTTCCACTGAATCC-3′) and inserted into pET-28a (+)-His vector (Sango Biotech, Shanghai, China). The inserted DNA fragments were sequenced on an ABI 3730 X 1 DNA Analyzer (Applied Biosystems, Foster City, CA, USA) by Sango Biotech Co. Ltd. Purified plasmid DNAs were prepared with a TIANprep Mini Plasmid Kit (TIANGEN, Beijing, China). The recombinant protein was affinity-purified by using a Ni-Sepharose column (Sango Biotech) as described previously (17). Recombinant PvTRAg-26 was then denatured with β-mercaptoethanol in sample buffer and analyzed by 13% sodium dodecyl sulfate-polyacrylamide gel electrophoresis (SDS-PAGE), followed by immunoblotting assay with an anti-His tag antibody (Qiagen, Hilden, Germany).

### Animal Immunization With Recombinant PvTRAg-26

Female BALB/c mice, 6-8 weeks old, were purchased from Vital River Laboratory Animal Technology Co, Ltd (Beijing, China). The mice were treated following the Guidelines for the Care and Use of Research Animals established by Anhui Medical University. Two groups of mice, 5 in each, were immunized subcutaneously (SC) with 50 μg of PvTRAg-26 in phosphate-buffered saline (PBS) or Freund's complete adjuvant (Sigma-Aldrich, San Francisco, CA, USA), for four times in a 3-wk interval. Boost injections were given after 3, 6, and 9 weeks of the priming with the same amount of antigen together with Freund's incomplete adjuvant (Sigma-Aldrich). The mouse sera were collected 2 weeks after the final boost and antibodies against PvTRAg-26 were measured as described previously (19).

### Enzyme-Linked Immunosorbent Assay (ELISA)

To investigate the prevalence of IgG subclasses against PvTRAg-26, serum samples from 35 *P. vivax*-infected patients and 15 uninfected individuals were selected. The ELISA was performed following the manufacture's instructions. Briefly, 5 μg/mL of PvTRAg-26 in coating buffer (0.05 M NaHCO_3_, pH 9.6) was incubated in 96-well ELISA plates (Corning-Costar, Corning, NY, USA) overnight at 4°C. The plates were incubated with 5% skimmed milk in PBS/T (0.05% Tween-20) for 1 h at 37°C to block nonspecific binding sites, and then incubated with 100 μL of individual sera diluted 1:50 in PBS/T. For IgG subclasses, the plates were washed and then incubated with horseradish peroxidase (HRP)-conjugated anti-human IgG1, IgG2, IgG3, and IgG4 antibodies (ImmunoWay, Plano, TX, USA) in dilution of 1:2000 in PBS/T. Chromogenic reactions were developed and measured based on the previous description (19).

To identify and compare the levels of total and subclasses of IgG antibodies against PvTRAg-26 in immunized mice sera, we coated ELISA plates with the recombinant antigen (1.25 μg/mL). The plates were blocked and incubated with mouse sera (1:2000 dilution in PBS/T) at 37°C for 45 min. For total IgG antibody measurements, the plates were washed and then incubated with HRP-conjugated anti-mouse IgG (H + L) (Invitrogen, Waltham, MA, USA) at a 1:50,000 dilution at 37°C for 45 min, whereas for IgG subclasses the plates were incubated with HRP-conjugated anti-mouse IgG1 (Invitrogen, MA, USA), IgG2a (Invitrogen), IgG2b (Abcam, Cambridge, MA, USA), and IgG3 (Abcam) antibodies at 1:30,000, 1:1000, 1:2000, and 1:1000 dilutions, respectively. Chromogenic reactions were developed and determined as previously described (19).

### Splenocyte Proliferation and Cytokine Assays

Spleens were removed from mice 2 weeks after the fourth immunization. Splenocytes obtained from PvTRAg-26 immunized or control mice were resuspended at concentrations of 5 × 10^6^ cells/mL in complete RPMI 1640 supplemented with 10% FBS. One hundred microliters of the cell suspension, and 100 μL of PvTRAg-26 proteins were added to 96-well culture plates at final concentrations of 2.5, 5, 10, or 20 μg/mL, respectively. Concanavalin A (Con A; Sigma-Aldrich) or lipopolysaccharide (LPS; Sigma-Aldrich) at final concentrations of 5 μg/mL or 10 μg/mL were used as positive control and PBS, as negative control. After a 72 h culture (37°C and 5% CO_2_), 100 μL of supernatants per well was collected and stored at −20°C for cytokine assays. Viable cells were measured using a Cell Counting Kit-8 (CCK-8 or WST-8) assay following the commercial kit protocols. Cytokines of interferon (IFN)-γ, interleukin (IL)-2, IL-4, and IL-10 were examined in culture supernatants of immunized mice using BD Cytometric Bead Array (CBA) Flex Set kit (BD Biosciences, San Diego, CA, USA) according to the manufacturer's instructions. The results were obtained by flow cytometry (FACS Calibur, BD Biosciences, San Jose, CA, USA) and analyzed using Flow Cytometric Analysis Program (FCAP) array software (Soft Flow, Kedves, Hungary).

### Indirect Immunofluorescence Assay (IFA)

IFA was performed with 4% paraformaldehyde-fixed parasites (17). Slides were incubated with the following primary antibodies: mouse anti-PvTRAg-26 sera (1:100) and rabbit anti-PvPHIST/CVC-81_95_ sera (1:100) or mouse anti-PvTRAg-26 sera (1:100) and rabbit anti-Band 3 antibody (1:200). After primary antibody reactions, the samples were then treated with secondary antibodies, Alexa Fluor 488-conjugated goat anti-mouse IgG (1:500, Invitrogen) or Alexa Fluor 568-conjugated goat anti-mouse IgG (1:500, Invitrogen), and 4, 6-diamidino-2-phenylindole (DAPI) (1:1000, Invitrogen) was used to stain the nuclei. The slides were then mounted with Prolong Gold anti-fade reagent (Invitrogen), and visualized with confocal laser-scanning microscopy (FV1000; Olympus, Tokyo, Japan) under oil immersion. Images were edited using Adobe Photoshop CS5 (Adobe Systems, San Jose, CA, USA).

### Statistical Analysis

Data were analyzed using GraphPad Prism (GraphPad Software, San Diego, CA, USA). Student's *t*-tests were used for comparing the difference between the means in each group. *P* < 0.05 was considered statistically significant.

### Ethical Considerations

The protocols of the study were approved by and carried out following the recommendations of the Life Ethics Committee of Anhui Medical University (No. 20160118) and the Animal Ethics Committee of Anhui Medical University (LLSC20160161). All subjects gave their written informed consents as per the Declaration of Helsinki.

## Results

### Expression of Recombinant PvTRAg-26

**Figure 1:**
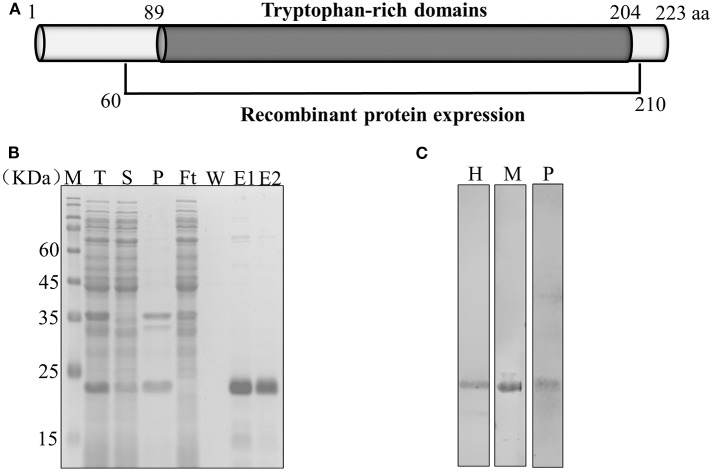
Schematic diagram showing the expression of *Pv*TRAg-26. **(A)** Diagram of the gene structure of *pvtrag*-26; aa, amino acids. **(B)** Expression and purification of recombinant PvTRAg-26. T: total translation mix, S: supernatant, P: precipitate, Ft: flow through, W: wash; E: elution treated with reducing buffer. **(C)** Recombinant PvTRAg-26 protein under reducing conditions was probed with an anti-His tag antibody, *P. vivax* malaria patient serum and immune mouse serum. H: anti-His tag antibody, M: immune mouse serum, P: *P. vivax*-infected patients' pooled sera.

The complete PvTRAg-26 (PlasmoDB accession No. PVX_112660) protein sequence in the sal-1 strain consists of 223 amino acids (26 kDa), rich in tryptophan residues (5.8%). The entire exon-2 (450 bp, encoding the tryptophan-rich domain) of *pvtrag-26* was amplified, cloned, and expressed in *Escherichia coli* (Figure 1A). The recombinant protein was purified under non-denaturing conditions, as shown in Figure 1B. The corresponding immunoblots were probed with an anti-His tag monoclonal antibody. Sera of *P. vivax*-infected patients and PvTRAg-26-immunized mice revealed a similar and specific migration pattern in PvTRAg-26 blotting (Figure 1C). Serum samples from uninfected individuals or normal mice were used as negative controls (data not shown).

### IgG Subclasses Recognizing PvTRAg-26 in Malaria Patients

**Figure 2:**
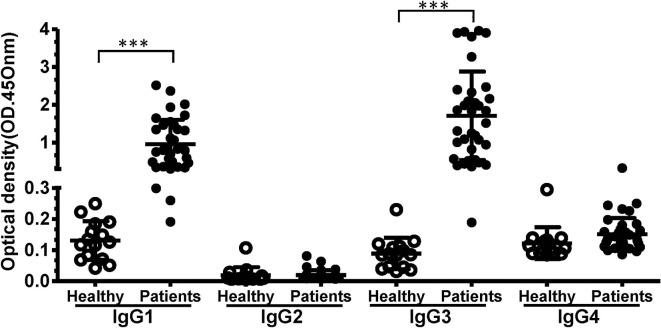
The levels of IgG subclasses recognizing PvTRAg-26 in sera from *P. vivax*-infected patients and uninfected individuals. Differences between IgG subclass levels in the negative and positive groups were analyzed using Student's *t*-tests. ***P < 0.001. The *P* values for IgG1 and IgG3 were <0.001, and those for IgG2 or IgG4 were >0.05. *P* < 0.05 was considered to indicate a significant difference.

We evaluated the prevalence of each IgG subclass antibodies against PvTRAg-26. The results showed that the mean levels and the hierarchy of IgG subclasses were as follows: IgG3 > IgG1 > IgG4 > IgG2 (Figure 2). IgG1 and IgG3 subclasses were the predominant antibodies compared to the others (*P* < 0.0001). Titres of the cytophilic antibodies (IgG3 and IgG1) were significantly higher than the non-cytophilic antibodies (IgG2 and IgG4) (*P* < 0.05). The concentrations of IgG1 and IgG3 in *P. vivax* patients were markedly elevated in comparison with those of uninfected controls.

### Anti-PvTRAg-26 IgG and Its Subclasses in the Sera of Immunized Mice

**Figure 3:**
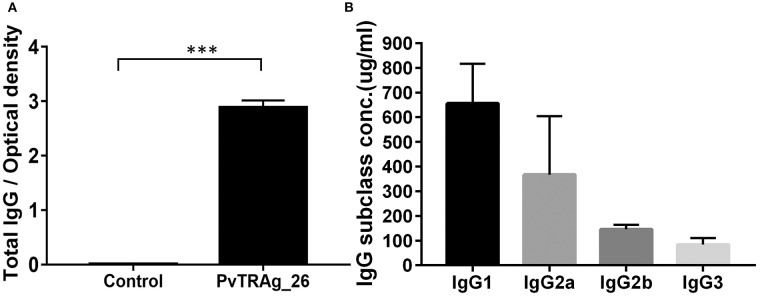
Levels of serum IgG and IgG subclasses in immunized mice. **(A)** Antigen-specific IgG levels were detected by ELISA in the sera of mice, as indicated after the final immunizations with PvTRAg-26, ***P < 0.001. **(B)** Serum IgG subclass pattern in PvTRAg-26-immunized mice, ***P < 0.001.

The serum levels of antigen-specific total IgG and its subclasses in response to PvTRAg-26 were determined. The level of total PvTRAg-26 specific IgG antibodies in antigen-immunized mice was notably increased in comparison with that in the control (*P* < 0.001) (Figure 3A). IgG1 subclass dominated compared to IgG2b and IgG3 in the immunized mice (Figure 3B).

### Antigen-Specific Immune Cell Responses and Cytokine Release

**Figure 4:**
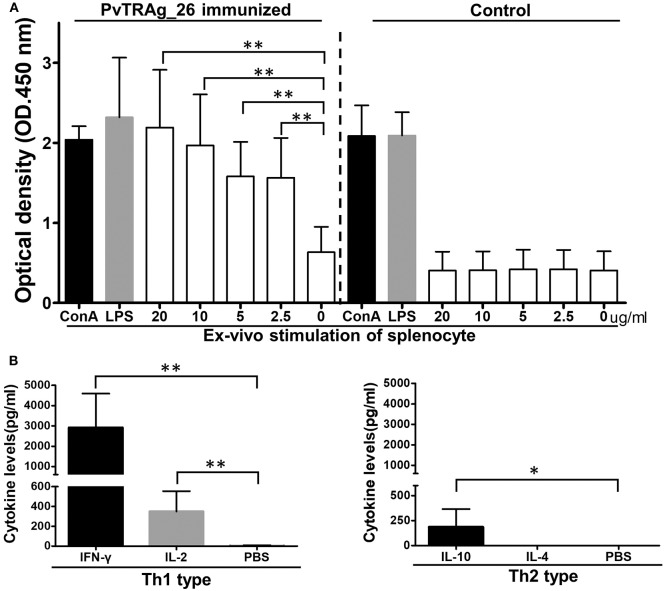
Proliferation index and cytokine secretion of splenocytes from mice immunized with PvTRAg-26. **(A)** Splenocytes from mice immunized with or without PvTRAg-26 were stimulated with various concentrations of PvTRAg-26 for 72 h before testing, as indicated. The splenocytes were stimulated with Con A or LPS as positive controls, as indicated, *P < 0.05, **P < 0.01. **(B)** Cytokine secretion profile of the splenocytes from the antigen-immunized mice.

Splenocyte proliferation assays and CBAs were performed to assess the antigen-specific response and the secretion of cytokines in immunized BALB/c mice. Splenocytes from the animals immunized with PvTRAg-26 and controls were stimulated with various concentrations of the PvTRAg-26 antigen, Con A, or LPS for 72 h. The cultural supernatants were harvested for CBA and cell proliferation assay. The splenocyte proliferation in PvTRAg-26-immunized mice showed a notable proliferative response compared to those of the control group (*P* < 0.01, Figure 4A). Additionally, cytokine determinations demonstrated a biased Th1-type response, with an elevated level of IFN-γ and IL-2 secretions in the splenocytes of mice immunized with PvTRAg-26. By contrast, production of Th2-type cytokines of IL-4 and IL-10 were remarkably dampened (Figure 4B).

### Association of PvTRAg-26 With CVC and the Erythrocyte Membrane in the Early Ring Stage

**Figure 5:**
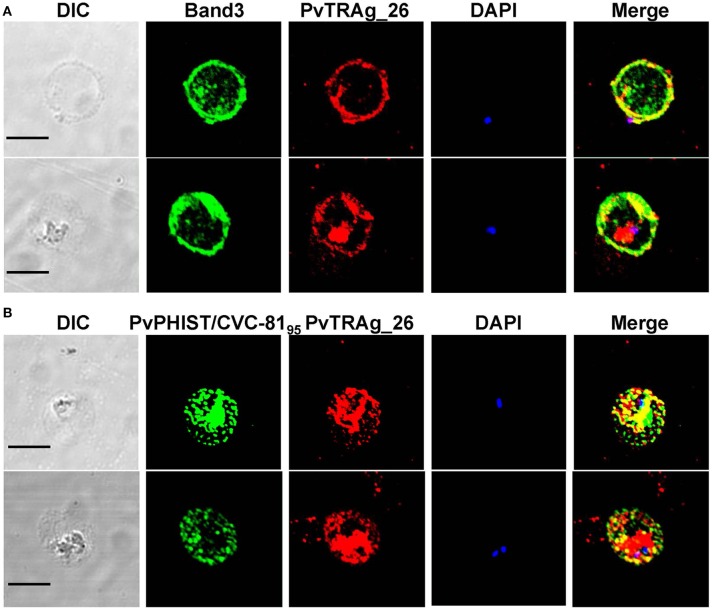
Localization of PvTRAg-26 in the early ring/trophozoite stage. **(A)** Ring stage parasites were double-labeled with mouse antisera against PvTRAg-26 (red) and a rabbit anti-Band 3 antibody (green). **(B)** Ring/trophozoite stage parasites were dual-labeled with mouse antisera against PvTRAg-26 (red) and rabbit antisera against PvCVC81_95_ (CVC marker, green). Nuclei were visualized with DAPI in merged images. The bar represents 5 μm.

Immunofluorescent assay was conducted by using anti-Band 3 (an erythrocyte membrane marker) and anti-PvPHIST/CVC-81_95_ (a CVC marker) sera. In the early ring or trophozoite stage of the parasite, PvTRAg-26 signals were merged (at least partially) with Band 3 and PvPHIST/CVC-81_95_. Specific fluorescence was visualized on the parasitophorous vacuolar membrane (PVM) (Figure 5) but the pre-immunized mouse sera did not show any signals (data not shown).

## Discussion

The TR-Ags of different *Plasmodium* species have been noted to have potential in malaria vaccine candidate screening (12, 16, 20–22) due to their parasite growth inhibition activity (23–25). *P. vivax* contains more abundant TR-Ags than any other human malaria parasites. Among them, PvTRAg-26, which contains positionally conserved tryptophan residues in a TR domain, could elicit a high level of protective IgG antibodies even in low malaria-endemic areas (17). Studies demonstrated that PvTRAg-26 possesses erythrocyte-binding ability (16). However, its immunogenic properties have not been fully explored. Here, we analyzed the cellular and humoral immune responses to PvTRAg-26 antigen in patients and immune mouse serum samples.

Antibodies play a crucial role in mediating acquired immunity to malaria during the intra-erythrocytic development stage of the parasites (26). The limited polymorphism of PvTR-Ags may contribute to their high immunogenicity. Our previous study exhibited that five PvTR-Ags, including PvTRAg-26, produced an elevated level of IgG antibodies in the sera of vivax patients (17). Definition of the IgG subclass response to PvTRAg-26 is important because the function of immune effectors varies in different subclasses (27, 28). Investigation of the antibody subclass response may provide further insight into the functions of antibodies and their roles in immune protection. Previous studies reported that cytophilic subclasses of IgG1 and IgG3 promote opsonic phagocytosis of merozoites or neutrophil-mediated killing, inducing a protection from malaria (29–31). Similarly, we noted that cytophilic antibody subclasses, IgG1 and IgG3, were predominant in host response to PvTRAg-26 antigen stimulation. Augmentation of humoral immune response mediated by IgG1 and IgG3 antibodies has been believed to play a pivotal role in reducing the risk of clinical malaria and parasitemia (32). During this process, complement activation mediated by IgG1 and IgG3 would be essential for inhibition of parasite invasion to host erythrocytes (33). Thus, further studies are needed to elucidate the functional activities of antibodies and their relationship with host protective immunity to vivax malaria.

Immunization with recombinant PvTRAg-26 resulted in a high level of IgG antibody response in mice, in which IgG1 subclass was predominant, followed by IgG2a. Similar results were also seen in the studies of mice with other malaria vaccine candidates, such as PvMSP1_19_, PfMSP1_19_, and PvMSP9 (34–36). The IgG1 and IgG3 antibodies are non-cytophilic and responsible for Th2-biased response (37), while the IgG2a and IgG2b are cytophilic and link to Th1 response in mice. Importantly, IgG2a antibodies in mice are considered to be most efficacious in complement activation and in activation of antibody-dependent cellular cytotoxic mechanism, in addition to ameliorating parasitemia caused by *P. yoelii* (31). We speculate that PvTRAg-26 antigen may induce a comprehensive Th1-Th2 protective response. Other studies have also shown the protective immunity in the presence of elevated levels of IgG1 and IgG2a with a combined Th1-Th2 immune responses to *P. yoelii* infection (38–44).

The mechanism of cellular immunity is closely associated with activation of phagocytes, antigen-specific cytotoxic T-lymphocytes and release of cytokines against infectious protozoan parasites (45). Cytokines are generally responsible for direct or indirect restriction of pathogenesis of infectious diseases (15, 46–48). It has been known that CD4^+^ T cells play a crucial role in protection against *Plasmodium* infection both in humans and in animals (49–51). The phenotype indicators of CD4^+^ T cells mainly include IFN-γ and IL-2 for Th1 response, and IL-4 and IL-10 for Th2 response (52, 53). Several studies demonstrated that PvTR-Ags elicit a combined Th1 and Th2 response in vivax malaria patients (51, 54). Here, we also observed a simultaneous up-regulation of cytokines of Th1 (IFN-γ and IL-2) and Th2 (IL-10) types, suggesting a systemic immune response of mice to PvTRAg-26 stimuation. The Th1 cytokines, e.g., IFN-γ, play an essential part in controlling malaria parasitemia during the early stages of infection (48, 55) and provide host with an effective protection from malaria (56, 57). IL-2 is a crucial T cell cytokine associated with proliferation, homeostasis, and differentiation of CD4^+^ and CD8^+^ T cells (58), and regulates the balance between effector Th1 cells and regulatory T cells in control of blood-stage malaria infection (59, 60). Contrarily, IL-10, a Th2 type cytokine, is known to be able to modulate the immune response to malaria parasites and to be involved in deterioration of parasitemia in *Plasmodium* infection (61, 62).

As visualized by IFA, PvTRAg-26 was transported from the parasite to the erythrocyte membrane through the CVC structure in the early ring stage. PvTRAg-26 was detectable on the PVM and its signal might be merged with Band 3 and CVC proteins. The function of the CVC largely remains unknown. It is hypothesized that the CVC may link to the transportation of materials from the parasite to the outside medium through the red blood cell cytoplasm (63–65). Similar to binding of PypAg-1/PypAg-3 to the membrane of red blood cells in rosette formation of *P. yoelii* (12), the co-localization of PvTRAg-26 with the CVC on the surface of infected erythrocytes suggests the transportation of PvTRAg-26 to the surface of the host cells, which may help promote the invasion process of *P. vivax* parasites. Further approaches *in vivo* are needed to determine the efficacy of PvTRAg-26 as a promising vaccine candidate.

## Conclusions

PvTRAg-26 possesses high antigenicity and immunogenicity and can induce potent Th1 and Th2 responses in patients and immunized mice. The recombinant PvTRAg-26 antigen has the potential in development of a novel molecular vaccine for prevention of *P. vivax* infection.

## Data Availability Statement

All datasets generated for this study are included in the article/supplementary material.

## Ethics Statement

The studies involving human participants were reviewed and approved by the Ethics Committee of Anhui Medical University (20160118), Anhui, China. The patients/participants provided their written informed consent to participate in this study. The animal study was reviewed and approved by the Animal Ethics Committee, Anhui Medical University (LLSC20160161).

## Author Contributions

BW, YX, and E-TH conceived and designed the experiments. LF, JX, and MZ designed the research protocol and performed the experiments. LF, JS, HX, and QF performed data acquisition and analysis. LF, JX, BW, YX, JS, J-HH and E-TH contributed to the interpretation of results and assisted in writing the manuscript. All authors read and approved the final manuscript.

## Conflict of Interest

The authors declare that the research was conducted in the absence of any commercial or financial relationships that could be construed as a potential conflict of interest.

## Abbreviations

*P. vivax*: *Plasmodium vivax*
TRAg: tryptophan-rich antigen
CVC: caveola-vesicle complex
PCR: polymerase chain reaction
PBS: phosphate-buffered saline
SDS-PAGE: sodium dodecyl sulfate-polyacrylamide gel electrophoresis
ELISA: enzyme-linked immunosorbent assay
Con A: concanavalin A
LPS: lipopolysaccharide
IFN-γ: interferon-γ
IL: interleukin
IFA: indirect immunofluorescence assay
PHIST: helical interspersed subtelomeric
PVM: parasitophorous vacuolar membrane.
